# High-dose methotrexate, thiotepa, orelabrutinib combined with or without rituximab in primary or secondary central nervous system diffuse large B-cell lymphoma: a single-center retrospective analysis

**DOI:** 10.7150/jca.85756

**Published:** 2023-09-25

**Authors:** Yufeng Li, Yajun Li, Ruolan Zeng, Yizi He, Liang Liang, Lijia Ou, Chang Su, Hui Zhou, Ling Xiao

**Affiliations:** 1 Central South University, Department of Lymphoma & Hematology, The Affiliated Tumor Hospital of Xiangya Medical School, Changsha, Hunan, China.; 2 Department of Histology and Embryology of School of Basic Medical Science, Central South University, Changsha, Hunan, China.

**Keywords:** bruton tyrosine kinase, orelabrutinib, central nervous system diffuse large B-cell lymphoma, ctDNA

## Abstract

**Purpose:** Central nervous system lymphoma (CNSL) is an aggressive non-Hodgkin's lymphoma (NHL) confined to the central nervous system (CNS). Orelabrutinib is an oral second-generation Bruton tyrosine kinase (BTK) inhibitor and a novel therapeutic strategy for CNSL. The purpose of this study was to evaluate the effectiveness and safety of high-dose methotrexate (HD-MTX), thiotepa, and orelabrutinib combined with or without rituximab (MTO±R)regimen in the treatment of patients with CNSL.

**Methods:** A total of 14 patients with CNS diffuse large B-cell lymphoma (DLBCL) were included in this retrospective study. All patients received the regimen MTO±R. Overall response rate (ORR), complete response rate(CR), partial response (PR), stable disease (SD), progressive disease (PD), progression-free survival (PFS), overall survival (OS), and the safety of MTO±R were assessed by the investigator.

**Results:** Fourteen patients were evaluable for safety, and 13 patients were evaluable for efficacy. The overall CR rate was 69.2%, and the ORR was 92.3% for total patients. For PCNSL, the CR rate and ORR were 55.6% and 88.9%, respectively. For relapsed/refractory CNSL, the CR rate and ORR were 66.7% and 91.7%, respectively. The median follow-up time was 12.8 months. The median PFS was 11.3 months, and the median OS was not achieved. The 12-month PFS and OS rates were 60% and 70%, respectively. Adverse events occurred in 17 cycles, and Grade 3 AEs occurred in 5 patients (35.7%).

**Conclusion:** MTO±R was an efficacious and well-tolerated regimen in patients with CNSL. A novel BTK inhibitor in combination with chemotherapy offers a new potential therapeutic strategy for patients with CNSL.

## Background

Central nervous system lymphoma (CNSL) is an aggressive non-Hodgkin's lymphoma (NHL) confined to the central nervous system (CNS), which includes brain parenchyma, spinal cord, cerebrospinal fluid, and eyes. It can be divided into primary central nervous system lymphoma (PCNSL) and secondary central nervous system lymphoma (SCNSL)^1^. Primary central nervous system lymphoma is rare, accounting for about 4% to 6% of NHL, and the aged are more likely to get it. Primary central nervous system lymphoma prevalence in the elderly increases yearly 2-5. Secondary CNSL(SCNSL) is the secondary central nervous system when it appears in patients with systemic lymphoma or recurs 6. The prognosis of both PCNSL and SCNSL is significantly worse than that of patients with non-central nervous system lymphoma 7. High-dose MTX is considered the cornerstone drug for treating patients with CNSL 8, and various combinations of chemotherapeutic drugs and HD-MTX have been investigated in multiple trials and found to significantly improve the prognosis of patients 9-12.

The B cell receptor (BCR) signaling pathway plays a key role in the pathogenesis of B cell lymphoma 13. Targeting components of the BCR signaling pathway, including Bruton tyrosine kinase (BTK), has made considerable progress in managing of refractory disease. BTK inhibitors combined with other chemotherapeutic drugs have shown good antitumor activity in a series of B cell lymphomas, such as mantle cell lymphoma, marginal zone lymphoma, and chronic B cell lymphoid leukemia 14-18. At the same time, as a first-generation (BTK) inhibitor, Ibrutinib shows single-drug activity in relapsed/refractory central nervous system (CNS) lymphoma 19, but PFS is short, and the CR rate is still not high enough. A meta-analysis has been conducted to report the good efficacy and tolerability of a first-generation BTK inhibitor therapeutic strategy, including Ibrutinib applied to CNSL 20, while clinical trials have shown that the combination of Ibrutinib, HD-MTX, and rituximab is well tolerated and safe 21. The second-generation BTK inhibitor zanubrutinib has also been reported to be effective in combination with other drugs in a retrospective study 22. As a second-generation BTK inhibitor, Orelabrutinib was approved in China in December 2020 for the treatment of MCL, CLL, and SLL patients who have received at least one chemotherapy treatment 23, and the second-generation BTK inhibitors are better tolerated than the first-generation BTK inhibitor Ibrutinib 14. It has been reported that, compared with Ibrutinib, Orelabrutinib exhibits better antitumor activity against diffuse large B-cell lymphoma and can enhance the antitumor activity of rituximab 24. Up till now, there are no reports about the efficacy and safety of second-generation BTK inhibitor Orelabrutinib combined with HD-MTX in CNSL.

In this study, we retrospectively analyzed 14 patients with central nervous lymphoma who were treated with high-dose methotrexate (HD-MTX), thiotepa plus orelabrutinib with or without rituximab (MTO±R), to evaluate its effectiveness and safety, thus providing some valuable information for the development of Orelabrutinib-based therapeutic regimens.

## Materials and Methods

### Patients

A total of 14 patients from Hunan Cancer Hospital from March 23, 2021, to September 19, 2022, were included in the retrospective study. All patients were diagnosed with PCNSL or SCNSL and received HD-MTX, Thiotepa, and Orelabrutinib(MTO) with or without rituximab (MTO±R). The inclusion criteria were as follows: (1). Age ≥18 years old and ≤75 years old; gender is not limited; (2). Primary CNS lymphoma (PCNSL) requires a histopathological diagnosis, and secondary CNS lymphoma (SCNSL) requires the histopathological diagnosis of diffuse large B-cell lymphoma (DLBCL); (3). Brain MRI or cranial CT showing evaluable solid lesions; (4). Patients with only meningeal lesions require CSF cytology to confirm lymphoma cells and/or imaging findings consistent with CSF; (4). ECOG score 0-2 points; (5). The expected survival time exceeds three months; The Exclusion criteria were as follows: (1). Patients with mental disorders, unstable systemic disease; (2). Patients with immunodeficiency or pregnancy.

### Treatment

All patients received a regimen of MTO±R: rituximab at 375mg/m^2^, D1; MTX at 3.5g/m^2^, D2; thiotepa at 40mg/m^2^, D2; Orelabrutinib at 150mg once daily until disease progression or intolerance; ervery 21 days per cycle. The use of rituximab depending on whether the patient benefits from rituximab (rituximab is used in patients younger than 60 years or who have undergone craniotomy). An interrupted or reduced dose was decided by a physician when patients experienced adverse events (AEs) (Table 1).

### Clinical assessment and follow-up

The treatment response was evaluated according to the Lugano 2014 Criteria for DBLCL and 2005 IELSG Criteria for PCNSL. The effectiveness of the treatment regimen was assessed by brain magnetic resonance imaging (MRI)/ brain contrast-enhanced computed tomography (CT)/ brain positron emission tomography PET-CT/Cytological results of CSF, bone marrow aspiration, and ophthalmology (every 2-3 cycles). Response evaluation includes partial response (PR), complete response (CR), progressive disease (PD), and stable disease (SD). Lymphoma is staged based on physical examination, whole-body computed tomography (CT), or PET-CT; progression-free survival (PFS) was calculated from the time of treatment initiation until disease progression or death. Overall survival (OS) was the time from treatment to death for any cause. The safety of the treatment regimen was evaluated based on physical examinations, vital signs, laboratory tests, and AEs. AEs are rated according to the generic terminology standard version 5.0. Cerebrospinal fluid (CSF) was collected from patients at baseline and after each treatment and sequenced by an MSK-HemePACT targeting panel, including 585 cancer genes specifically targeting genes related to hematological malignancies. See SHU et al. for genome analysis 25.

### Statistical analysis

The PFS and OS were estimated using the Kaplan-Meier method with 95% confidence intervals (CIs). All analyses were performed with data obtained until March, 2022, using SPSS software version 19.00.

## Results

### Patient characteristics

The baseline characteristics of the 14 patients are summarized in Table 2 and 3, including 7 males (n=7, 50%) and 7 females (n=7, 50%). The median age was 58 years, with 10 cases of PCNSL (n=10, 71.4%) and 4 cases of SCNSL (n=4, 28.6%). There were 13 cases of non-GCB type (n=13, 92.9%) and 1 case of GCB type (n=1, 7.1%). 2 patients developed deep intracranial lesions (n=2,14.3%), and 4 patients had LDH elevation (n=5, 35.7%). Ki-67 was measured in 13 patients, and Ki-67 was greater than or equal to 75% in 9 patients (n = 9, 69.2%). BCL-2 was detected in 12 patients, 11 of whom were positive (n=11, 91.7%). BCL-6 was positive in 13 patients (n=13, 100%). MUM-1 was positive in 14 patients (n=14, 100%).

Fourteen patients received a total of 60 cycles of the MTO±R regimen: 2 cases of 2 cycles (n=2,14.3%), 6 cases of 3 cycles (n=6, 41.2%), 1 case of 4 cycles (n=1,7.1%), 3 cases of 6 cycles (n=3,21.4%), and 2 cases of 8 cycles (n=2,14.3%).

The patients were followed up to September 19, 2022, with a median follow-up of 12.8 months.

### Treatment outcomes

**Figure 1:**
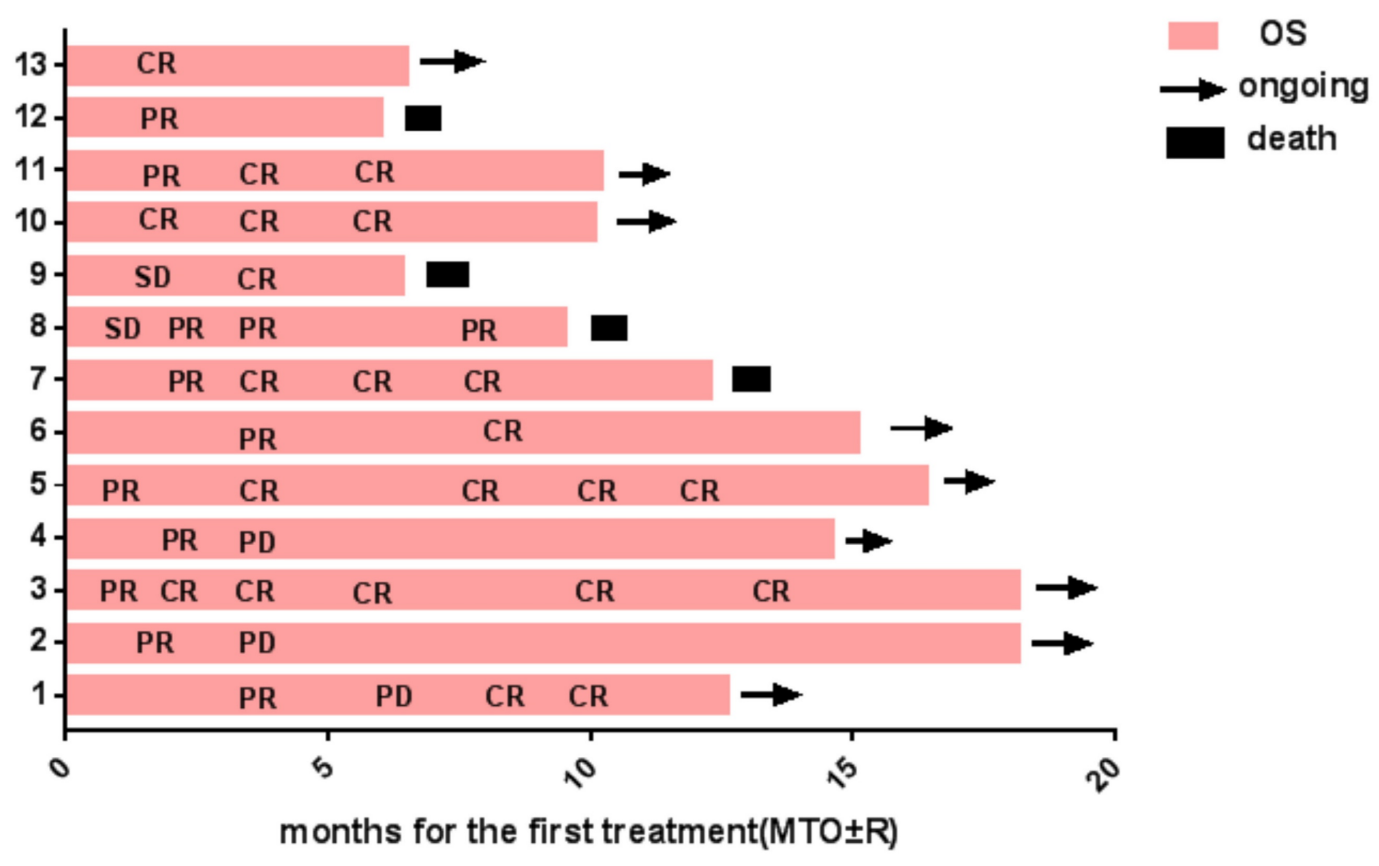
** Swimmer plots representing the durations of treatment in patients.** Time to response and duration of response. OS,overall survival CR complete response, PR partial response, PD progressive disease, SD stable disease.

Efficacy was evaluable in 13 of the 14 patients (Table 4); 1 patient has not been evaluated due to lack of data. Among the evaluable 13 patients, the overall CR rate was 69.2%, and the ORR was 92.3% for total patients (Figure 1). 1 patient was Newly diagnosed with CNSL, and 12 patients were Relapsed/refractory CNSL. Newly diagnosed CNSL included CR in 1 case (n=1, 100%), and ORR is 100%. The efficacy of 12 Relapsed/refractory CNSL patients was as follows: CR in 8 cases (n=8, 66.7%), PR in 3 cases (n=3, 25%), PD in 1 case (n=1, 8.3%), and ORR in 91.7%.

Among the 13 patients evaluated for efficacy, 9 were PCNSL, and 4 were SCNSL. PCNSL included CR in 5 cases (55.6%), PR in 3 cases (33.3%), and PD in 1 case (11.1%), ORR is 88.9%. Efficacy in 4 patients with SCNSL: CR in 4 patients (100%) and ORR in 100%. Overall, 13 evaluable patients had a CR rate of 69.2% and an ORR of 92.3%. According to the previous treatment history, 9 out of 12 patients with refractory recurrence achieved better efficacy after receiving the MTO±R regimen, which may mean that the MTO±R regimen is a more effective regimen for patients with refractory recurrence CNSL (Table 5).

**Figure 2:**
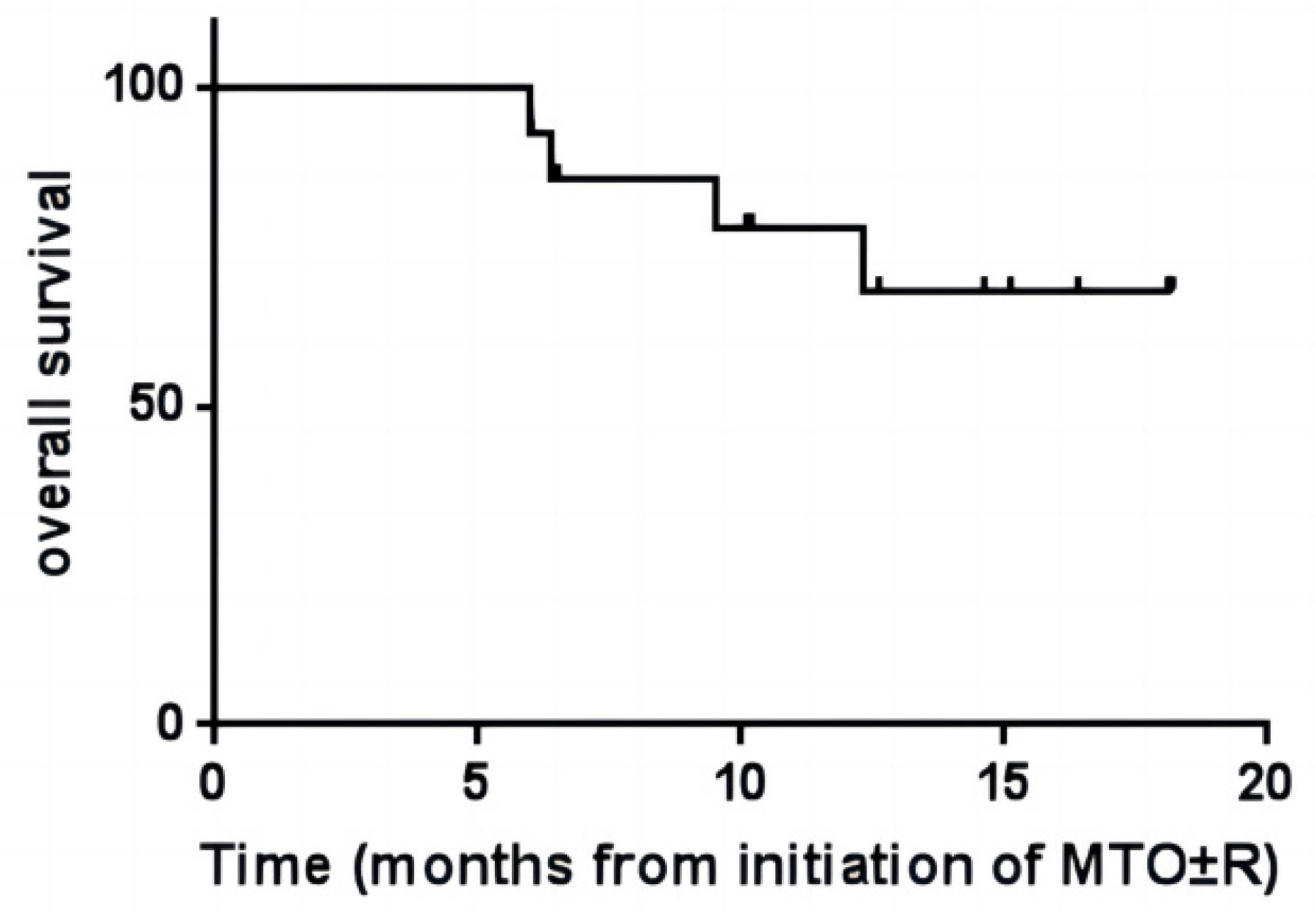
** Kaplan-Meier overall survival curves (OS).** Figure 2 shows the OS after the patient received MRO±R.

**Figure 3:**
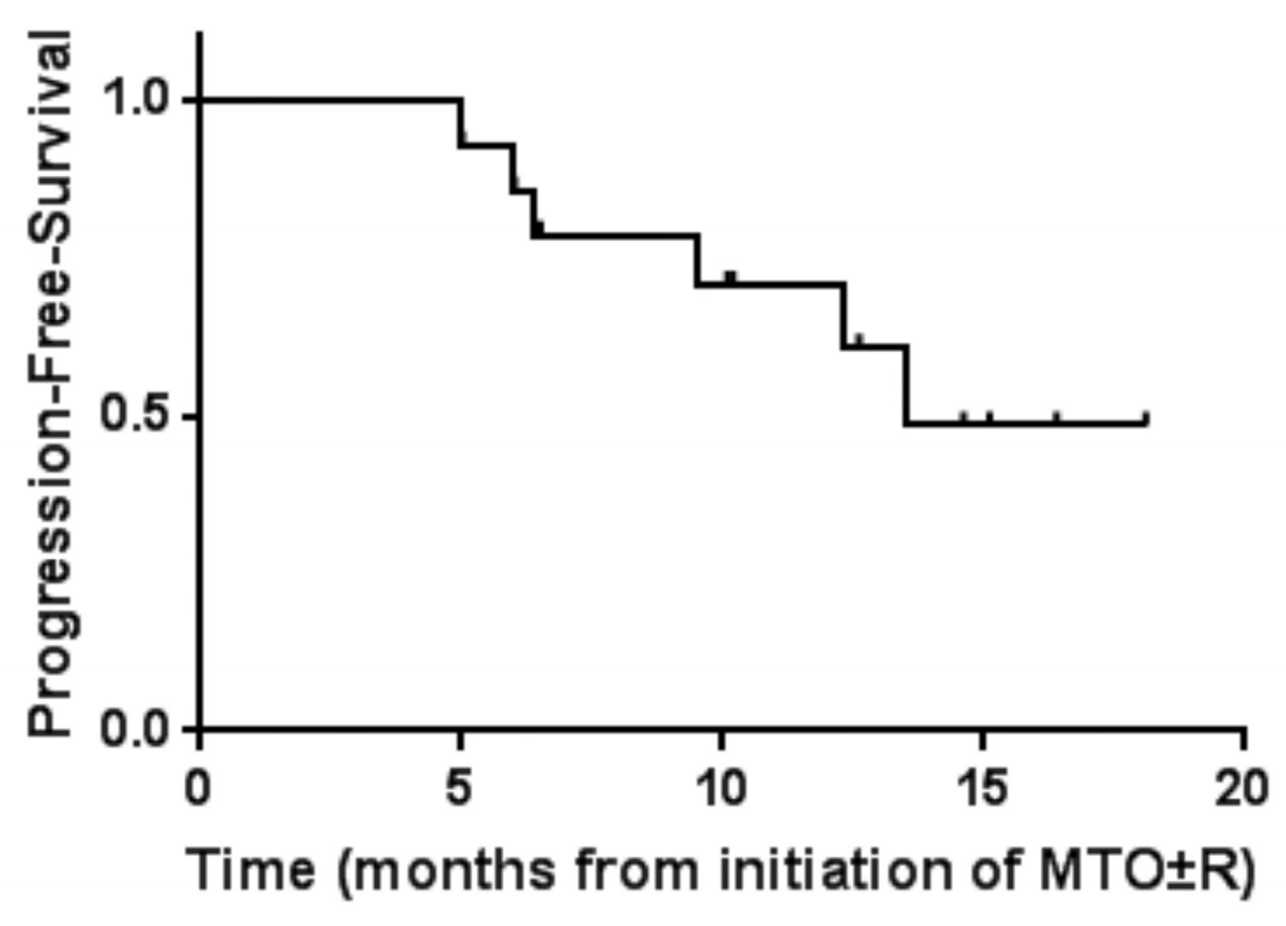
** Kaplan-Meier progression free survival curves (PFS).** Figure 3 shows the PFS after the patient received MRO±R.

As of September 2022, 14 patients with primary/secondary central nervous lymphoma have received a regimen of Orelabrutinib in combination with high-dose methotrexate and Thiotepa (MTO), 10 patients have received rituximab therapy, but 4 patients have not received rituximab therapy (Table 2). The median follow-up was 12.8 months, the median PFS was 11.3 months, and the median OS was not achieved (Figure 2 and 3). The 12-month OS rate was 70%, and the 12-month PFS rate was 60%. As of September 19, 2022, 4 patients died due to the failure of disease progression rescue.

**Figure 4:**
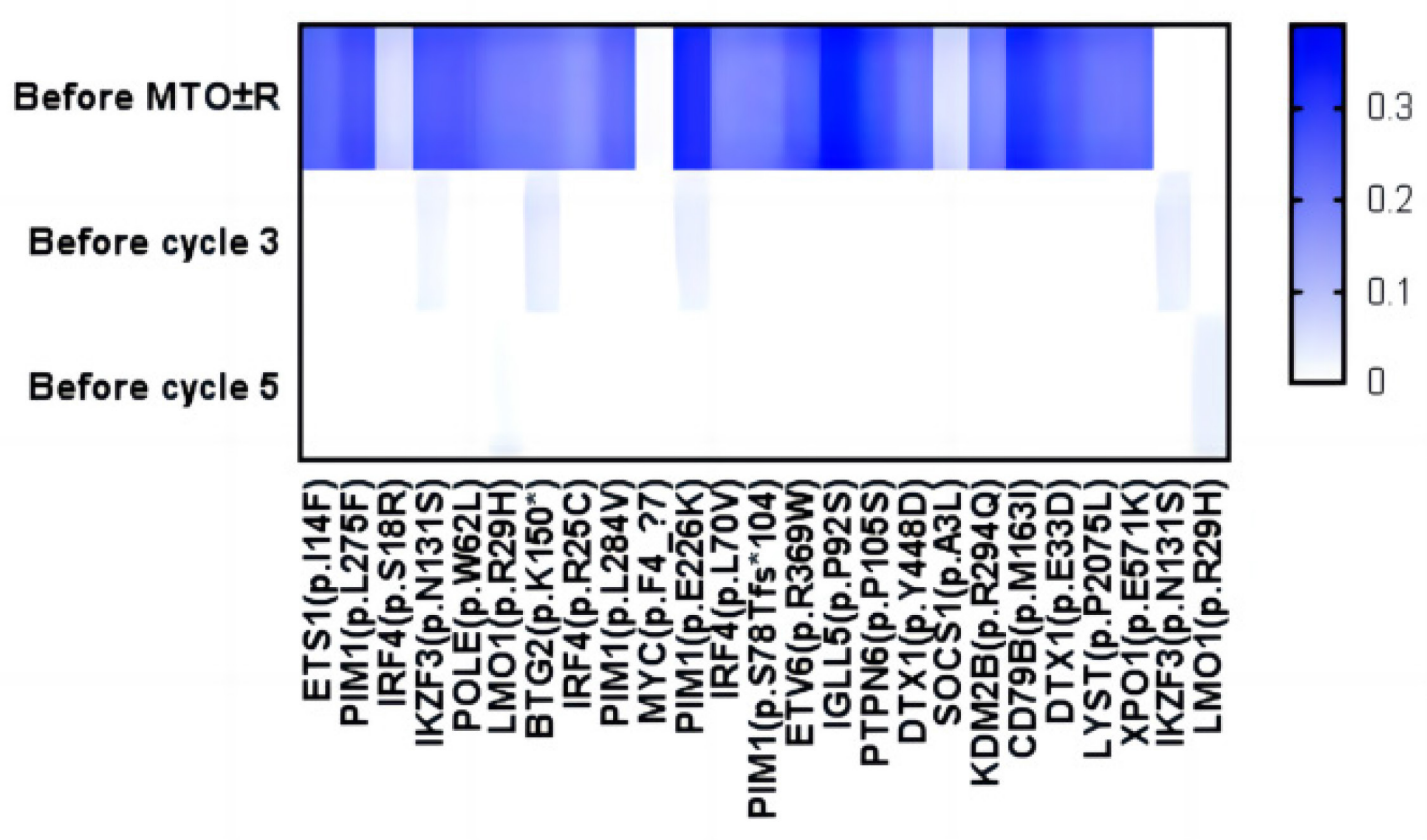
** Abundance changes of ctDNA.** Heat map of mutant allele frequencies of all mutations in CSF collected from patients before the start of MRO±R and during the combined treatment of MRO±R (C3, C5), with mutant allele frequency scale = 0 (white) or 1 (dark blue).

ctDNA was detected in one patient before and after treatment; the patient, whose ID was 11, received two cycles of R-MT therapy after admission, and the efficacy was evaluated as SD. Then, to improve the efficacy, R-MTO therapy was used. ctDNA detection was performed before using this regimen, and ctDNA detection was performed every two subsequent cycles after treatment. Respectively. After 2 cycles of treatment compared to pre-treatment, the gene abundance of each tumor mutation was reduced, and most tumor mutant genes were negative, such as PIM1 and CD79b (Figure 4). In addition, the patient's efficacy was assessed as CR, suggesting that ctDNA can be used as a biomarker in CNSL to monitor the efficacy of treatment regimens and evaluate the patient's prognosis.

### AEs

A total of 60 cycles of treatment were performed on 14 patients. Adverse events occurred in 17 cycles (Table 6). 14 cycles showed varying degrees of myelosuppression (n=14, 22.9%), mainly manifested as erythrocytopenia (n=1, 1.7%), leukopenia (n=11, 18.3%) and thrombocytopenia (n=4, 6.7%), of which two cycles were grade I myelosuppression (n = 2, 14.2%), 7 cycles were grade II myelosuppression (n=7, 50%), and 5cycles were grade III myelosuppression (n=5, 35.7%). The liver injury occurred in two cycles (n=2, 11.8%), transaminase increased in one cycle (n=1, 1.6%), and creatinine increased in one cycle (n = 1, 1.6%). Meanwhile, a patient developed a rash (n=1, 1.6%) after the initial administration of orelabrutinib, which disappeared after drug discontinuation. One patient died due to disease progression.

## Discussion

Central nervous system lymphoma is a very low incidence and is common in middle-aged and elderly patients. For PCNSL, it only accounts for 4%-6% of NHL, and only 4-5 people out of every 1 million are diagnosed with PCNSL 1. At the same time, its prognosis is generally poor. Studies have reported that the median survival time of PCNSL is 26 months, and the 5-year OS is 30% 26. The median survival time of SCNSL is less than 12 months 22, 27. Therapeutic regimens for CNSL have been continuously developing. High-dose methotrexate is considered the cornerstone of most induction regimens for treating CNSL 28-31. But HD-MTX alone or in combination with rituximab has been reported to have a CR rate of 36-73% and progression-free survival of 4.5-26.7 months 11. Because the curative effect of HD-MTX is not very satisfactory, more and more schemes containing HD-MTX have been put forward and studied by researchers, and the combination of some drugs has greatly improved the curative effect and safety of HD-MTX.Although the study of IELSG32 shows that rituximab combined with MTX+ cytarabine + thiotepa (MATRix) has higher ORR and survival rate than the other two regimens (MA, RMA) that do not contain thiotepa for the treatment of newly diagnosed PCNSL, the high advice events rate still limits the application of this regimen 32. In addition, the study of Fox et al. shows that the immuno-chemotherapy regimen (thiotepa+ifosfamide +etoposide+rituximab, TIER) that contains thiotepa in the treatment of relapsed and refractory PCNSL obtains 52% ORR with good safety 33. The above studies suggest that an immuno-chemotherapy regimen containing high-dose MTX and thiotepa in combination with rituximab may be a promising regimen for treating CNSL.

BCR signaling pathway plays an important role in CNSL 34. Bruton tyrosine kinase (BTK) connects B cell antigen receptor (BCR) and Toll-like receptor with NF-κB, and Systematic sequencing of PCNSL showed that CD79B, which accompanies the BCR signaling pathway of MYD88, mutates more frequently in PCNSL than in systemic DLBCL suggesting that BTK inhibitors may play an excellent anti-tumor role in CNSL 35. Currently, ibrutinib, zanubrutinib, and tirabrutib have relatively good therapeutic effects on CNSL. A PRISMA-Compliant Single-Arm Meta-Analysis showed that the ORR of ibrutinib in treating PCNSL was 72%, and the CR and PR rates were 53% and 22%, respectively 20. A Phase I/II study reported that treating R/R PCNSL by tirabrutib obtained an overall response rate of 64%, and the median progression-free survival was 2.9 months 36. Currently, clinical reports of Orelabrutinib in central nervous lymphoma are very rare. Our results show a surprising anti-tumor activity of a regimen based on Orelabrutinib for patients with central nervous lymphoma. In 13 patients with evaluable efficacy, the ORR was 92.3%, superior to the Ibrutinib-based regimen (ORR 55%) 36, 37. This result may be based on the good permeability of Orelabrutinib for the blood-brain barrier. Studies reported that the CSF concentration of Orelabrutinib was 20.10±14.70 ng/mL, much higher than 0.77 ng/mL of Ibrutinib 38. This may also be one of the advantages of this regimen compared with the regimen containing ibrutinib. To sum up, we adopted MTO±R scheme, hoping to bring better prognosis to patients through the novel second-generation BTK inhibitor Orelabrutinib.

In our study, 14 patients experienced AES in 17 of the 60 cycles of treatment and myelosuppression to varying degrees (n=14,23.3%) in 14 cycles, mainly manifested as erythrocytopenia (n=1,1.7%), leukopenia (n=11,18.3%) and thrombocytopenia (n=4,6.7%). The liver injury occurred in two cycles (n=2,3.3%), transaminase increased in one cycle (n=1,1.7%), and creatinine increased in one cycle (n = 1,1.7%). Meanwhile, a patient developed a rash on the low back (n=1,1.7%) after the initial administration of orelabrutinib. One patient died due to disease progression. One patient experienced symptom remission after dose reduction, and one patient experienced symptom disappearance after drug discontinuation. The adverse events of other patients were mild. All the AEs were relieved after corresponding treatment measures were given. Five patients had Grade 3 AES(n=5,35.7%), which was lower than the previous 39% reported for Ibrutinib 38; meanwhile, compared with the Grade 4 adverse reaction in the other study, no adverse reaction was found in this study 21. At the same time, the common adverse reactions of Ibrutinib, such as fever, pneumonia, neutropenia, and diarrhea, were rarely reported in this study. These data demonstrate that the second-generation BTK inhibitor, Orelabrutinib, has better significant efficacy and lower AEs rate than the first-generation Ibrutinib, which may be due to the high selectivity of Orelabrutinib.

ctDNA (circulating tumor DNA) is now a commonly used and clinically recognized alternative to biopsy. It is increasingly being used as a biomarker to guide clinical decision-making so as to detect metastatic foci better, monitor therapeutic effects and determine the best treatment method to minimize treatment costs and side effects 39, such as non-small cell lung cancer 40 and breast cancer 41. ctDNA has been used in many subtypes of lymphoma, including diffuse large B cell lymphoma, follicular lymphoma, mantle cell lymphoma, Hodgkin's lymphoma, peripheral T cell lymphoma, and primary central nervous system lymphoma 42-45. At the same time, central nervous system (CNS) malignant tumors may be difficult to diagnose, and many responses to existing therapies are unsatisfactory. Due to the difficulty and risk of brain biopsy and the low specificity and sensitivity of currently available minimally invasive methods, it may be challenging to monitor the treatment response and tumor recurrence of patients with CNS malignant tumors 46. At present, it has been shown that cerebrospinal fluid (CSF) circulating tumor DNA (ctDNA) can be used as a liquid biopsy to characterize and monitor CNS malignant tumors 45, and some studies have shown that CSF ctDNA may detect the existence of CNS recurrence and residual diseases earlier than traditional methods. Therefore, using ctDNA as a biomarker of CNSL to predict the therapeutic effect and prognosis has a very broad prospect 46; considering the economic pressure of patients, only one of the 14 patients in this study had a longitudinal ctDNA test. After the treatment, the tumor mutation abundance of this patient decreased obviously, and the tumor mutation gene turned negative, which was consistent with the results of a phase I B clinical trial of Ibrutinib in CNSL. In this clinical trial, the clearance of ctDNA in cerebrospinal fluid was detected after the use of Ibrutinib 21. At present, more and more CNSL patients are beginning to collect CSF to detect ctDNA instead of biopsy. In the follow-up, we will collect more patients who meet the standard for ctDNA detection and explore the relationship between Orelabrutinib and ctDNA and its influence on clinical prognosis.

Because our study is a single-center, non-randomized study, it has the following limitations. First, the number of cases is small. Second, the follow-up time is relatively insufficient. Third, treatment regimens based on Orelabrutinib are generally performed on the second line. Therefore, more studies with larger sample size and longer follow-up time are needed to verify the effectiveness and safety of MTO±R regimen.

## Conclusion

High-dose methotrexate (HD-MTX), thiotepa, orelabrutinib combined with or without rituximab (MTO±R) has been shown to be effective with low toxicity in the treatment of patients with CNSL. The appearance of Orelabrutinib may provide a new treatment option for patients with CNSL.

## Figures and Tables

**Table 1:** MRO±R regimens

Chemotherapy regimens	Drug dosage and usage	No. patients (%)
MTX+Thiotepa+BTKi	MTX at 3.5g/m^2^, D1 Thiotepa 40mg/m^2^, D1 Orelabrutinib at 150mg once daily	4 (28.5)
Rituximab+MTX+Thiotepa+BTKi	rituximab at 375mg/m^2^, D1 MTX at 3.5g/m^2^, D2 Thiotepa40mg/m^2^, D2 Orelabrutinib at 150mg once daily	10 (71.5)

**Table 2:** Baseline characteristics of included patients

Characteristics	No. patients	Percent %
**Gender**		
Male	7	50
Female	7	50
**Age (rank, median)**	36-74 (57.5)	
**CNSL**		
PCNSL	10	71.5
SCNSL	4	28.5
**B symptom**		
Yes	0	0
No	14	100
**Ann Arbor stage**		
ⅠⅡ	11	78.5
Ⅲ Ⅳ	3	21.5
**IELSG**		
1	1	7.1
2	8	57.2
3	4	28.6
4	1	7.1
**KPS**		
≤70	4	28.6
>70	10	71.4
**MSKCC**		
Low-risk	2	14.2
Median-risk	4	28.6
High-risk	8	57.2
**Deep intracranial lesion**		
Yes	12	85.7
No	2	14.3
**ECOG**		
0-1	9	64.3
2-3	5	35.7
**LDH**		
Normal	9	64.3
Elevated	5	35.7
**IPI**		
0-2	13	92.9
3-5	1	7.1
**Subtype of DLBCL(n=14)**		
GCB	1	7.1
Non-GCB	13	92.9

**Table 3:** Baseline characteristics of included patients 2

ID	Sex	Age	Ki-67 (%)	BCL-2	CD10	BCL-6	MUM-1	Cerebrospinal fluid protein	Deep intracranial lesions
1	M	74	50-60	1	0	1	1	normal	Yes
2	M	64	80	1	0	1	1	normal	Yes
3	F	58	85	1	0	1	1	elevated	Yes
4	M	63	90	1	0	1	1	NA	Yes
5	F	71	70	1	1	1	1	elevated	Yes
6	F	68	80	1	0	1	1	NA	Yes
7	M	57	90	1	0	1	1	NA	no
8	F	36	90	1	0	1	1	normal	Yes
9	F	64	90	1	0	1	1	NA	Yes
10	F	57	80	NA	1	1	1	normal	no
11	M	46	NA	NA	0	NA	1	normal	Yes
12	F	47	70	1	0	1	1	normal	Yes
13	M	56	70	1	0	1	1	elevated	Yes
14	F	52	80	0	0	1	1	elevated	Yes

In the table, 0 is negative, 1 is positive, and NA indicates that the patient was not tested

**Table 4:** Summary of efficacy data for therapy

Variable	Newly diagnosed CNSL (*n*=1)	Relapsed/refractory CNSL (*n*=12)	PCNSL (n=9)	SCNSL (n=4)	Total (*n*=13)
ORR%	100	91.7	88.9	100	92.3
CR/CRu	1	8	5	4	9
PR	0	3	3	0	3
SD	0	0	0	0	0
PD	0	1	1	0	1
12-month PFS%	-	60	83.3	50	60
12-month OS%	-	70	83.3	50	70
Duration of orelabrutinib treatment (months)	10.2	12.8 (6.5-18.2)	13 (6.5-18.2)	12 (7-13)	12.8 (6.5-18.2)

**Table 5:** Previous treatment history of included patients

ID	CNSL	Diagnosed	Previous treatment regimen	Previous therapeutic effect	Regimen	Therapeutic effect
1	SCNSL	Relapsed	RCHOP	PD	R-MTO	CR
2	PCNSL	refractory	MT	SD	MTO	PD
3	PCNSL	refractory	MT	PR	R-MTO	CR
4	PCNSL	refractory	R-MT	PR	R-MTO	NA
5	PCNSL	refractory	MT	PR	MTO	PR
6	PCNSL	refractory	MT	PR	MTO	CR
7	PCNSL	refractory	MT	PR	R-MTO	PR
8	SCNSL	refractory	RCHOP	PR	R-MTO	CR
9	PCNSL	Relapsed	temozolomide	SD	MTO	PR
10	SCNSL	Relapsed	R-ICE	SD	R-MTO	CR
11	PCNSL	refractory	R-MT	SD	R-MTO	CR
12	PCNSL	Newly	NA	NA	R-MTO	CR
13	SCNSL	Relapsed	radiotherapy+RCHOP	SD	R-MTO	CR
14	PCNSL	Relapsed	Gamma knife treatment	NA	R-MTO	CR

RCHOP, rituximab+cyclophosphamide+doxorubicin+vincristine+prednisone; MT,HD-MTX+Thiotepa; R-MT, rituximab+HD-MTX+Thiotepa; R-ICE, rituximab+ifosfamide+carboplatin+etoposide

**Table 6:** AEs in all cycles (n=60)

	All grades, (n, %)	Grade1-2, (n, %)	Grade 3-4, (n, %)
Any AEs	17 (28.3)		
Any AEs leading to discontinuation	0		
Any AEs leading to interruption	1 (1.7)		
Any AEs leading to dose reduction	1 (1.7)		
Hematologic events			
Erythrocytopenia	1 (1.7)	1 (1.7)	
Thrombocytopenia	4 (6.7)	2 (3.3)	2 (3.3)
Leukopenia	11 (18.3)	7 (11.7)	4 (6.7)
Non-hematologic events			
Alanine aminotransferase increased	1(1.7)	1 (1.7)	
Creatinine increased	1(1.7)	1 (1.7)	
rash	1 (1.7)	1 (1.7)	
